# New Constituents from the Rhizomes of Egyptian *Iris germanica* L.

**DOI:** 10.3390/molecules17032587

**Published:** 2012-03-02

**Authors:** Sabrin R. M. Ibrahim, Gamal A. Mohamed, Nawal M. Al-Musayeib

**Affiliations:** 1 Department of Pharmacognosy, Faculty of Pharmacy, Assiut University, Assiut 71526, Egypt; 2 Department of Natural products, Faculty of Pharmacy, King Abdulaziz University, Jeddah 211589, Saudi Arabia; 3 Department of Pharmacognosy, Faculty of Pharmacy, King Saud University, Riyadh 11451, Saudi Arabia

**Keywords:** *Iris germanica* L., Iridaceae, irigenin S, iriside A, antimicrobial, anti-inflammatory

## Abstract

Chemical investigation of the methanolic extract of the rhizomes of *Iris germanica* L. (Iridaceae) afforded two new compounds; irigenin S (**7**) and iriside A (**12**), together with ten known compounds: stigmasterol (**1**), α-irone (**2**), γ-irone (**3**), 3-hydroxy-5-methoxyacetophenone (**4**), irilone (**5**), irisolidone (**6**), irigenin (**8**), stigmasterol-3-*O*-*β*-D-glucopyranoside (**9**), irilone 4'-*O*-*β*-D-glucopyranoside (**10**) and iridin (**11**). Their structures were established by UV, IR, 1D (^1^H and ^13^C) and 2D (^1^H-^1^H COSY, HMQC, and HMBC) NMR spectroscopy, in addition to mass spectroscopic data and comparison with literature data. The methanolic extract was evaluated for its antimicrobial activity. Both the methanolic extract and the isolated flavonoids were tested for their anti-inflammatory activity.

## 1. Introduction

**Figure 1:**
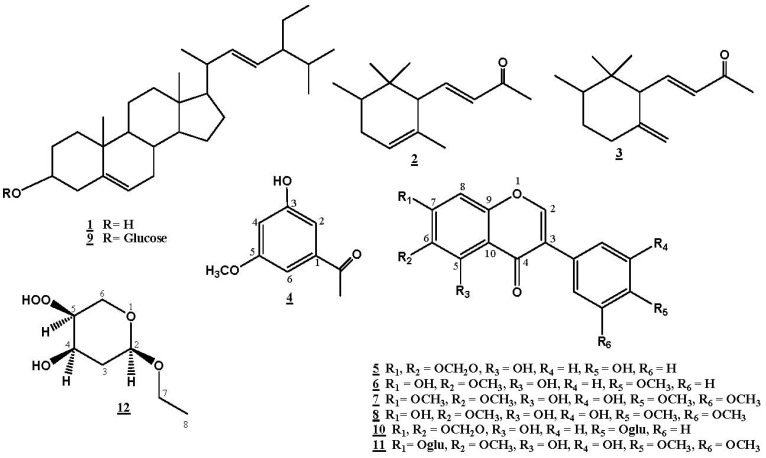
Structures of isolated compounds.

The genus *Iris* belongs to the family Iridaceae, which comprises over 300 species [1]. *Iris* species have an immense medicinal importance and are used in the treatment of cancer, inflammation, bacterial and viral infections [2,3]. The compounds isolated from these species were reported to have piscicidal, anti-neoplastic, antioxidant, antitumor, anti-plasmodial, molluscicidal, and anti-tuberculosis properties, in addition to protein kinase C activation activity [4,5,6,7,8,9,10]. *Iris germanica* L., known as Irsa, is widely distributed in most parts of the World, and also cultivated as an ornamental plant [11,12,13]. The essential oil of *I*. *germanica* rhizomes is used in perfumes and cosmetics, while the leaves are a rich source of ascorbic acid and vitamins [1,14]. The aqueous extract of *Iris germanica* decreases smooth muscle activity *in vivo*, stimulates respiration, and shows central anti-serotonin activity. It also induces a transitory hypotension accompanied by a negative inotropic effect [11,12]. A root decoction of the plant has been used as an antispasmodic, anti-inflammatory, emmengogue, stimulants, diuretic, aperients, and violently cathartic [11,12,15]. *Iris germanica* is considered as a rich source of secondary metabolites such as flavonoids [9,11,12,13,14,16,17,18,19,20], triterpenes [4,8,21,22,23,24,25,26], benzene and benzoquinones derivatives [15,19]. This article reports the isolation and characterization of two new compounds—irigenin S (**7**) and iriside A (**12**)—along with ten known compounds: stigmasterol (**1**) [27], α-irone (**2**) [23], γ-irone (**3**) [23], 3-hydroxy-5-methoxyacetophenone (**4**) [28], irilone (**5**) [17,29,30], irisolidone (**6**) [15,17,31], irigenin (**8**) [16,17,32], stigmasterol-3-*O*-*β*-D-glucopyranoside (**9**) [33], irilone 4'-*O*-*β*-D-glucopyranoside (**10**) [14] and iridin (**11**) [15,17,34] (Figure 1) from the rhizomes of *I. germanica* L. growing in Egypt, the antimicrobial activity of its methanolic extract, as well as anti-inflammatory activity of the methanolic extract and the isolated flavonoids.

## 2. Results and Discussion

Irigenin S (**7**) was isolated as yellow needles (MeOH), m.p. 192–193 °C. The UV spectrum of **7** showed absorption bands at λ_max_ 201, 265, and 323 nm, suggesting its isoflavone nature, further confirmed by the singlet signal at δ_H_ 8.42 for H-2 [29,35]. The IR spectrum showed absorption bands at 3437, 2956, 1651, and 1065 cm^−1^, indicating the presence of O-*H*, aromatic C-H, *α*,*β*-unsaturated carbonyl, and C-O functions in the molecule. Compound **7** possessed the molecular formula C_19_H_18_O_8_ by HRESIMS, that showed a pesudomolecular ion peak at *m/z* 375.1002 [M+H]^+^. The ^1^H-NMR spectrum showed resonances for 18 protons; three aromatic protons signals at δ_H_ 8.42 (s, H-2), 6.61 (s, H-8), 6.75 (d, *J* = 1.5 Hz, H-2', 6'), four methoxy group signals at δ_H_ 3.70 (4'-OCH_3_), 3.76 (6-OCH_3_), 3.79 (5'-OCH_3_), and 3.88 (7-OCH_3_), 3' and 5 chelated hydroxy groups at δ_H_ 9.31 and 12.97 (Table 1). 

**Table 1:** NMR data of compounds **7** and **12** (DMSO-*d*_6_, 500 and 125 MHz).

	7			12			
Position	δ_H_ (*J* in Hz)	δ_C_, mult.	HMBC	δ_H_ (*J* in Hz)	δ_C_, mult.	^1^H-^1^H COSY	HMBC
			H→C				H→C
1	-	-	-	-	-	-	-
2	8.42 s	154.8, d	1', 3, 4, 9	5.08 dd (2.0, 4.1)	103.2, CH	3	3, 4, 5, 6, 7
3	-	126.3, C	-	1.96 m	41.2, CH_2_	2, 4	2, 4, 5
				1.85 m			
4	-	180.7, C	-	4.09 t (4.5)	70.9, CH	3, 5, 3-OH	-
5	-	151.3, C	-	3.68 m	86.9, CH	4, 6	2, 4, 6
6	-	132.1, C	-	3.36 m	63.7, CH_2_	5	2, 4, 5
7	-	154.5, C	-	3.61 m	62.1, CH_2_	8	2, 3, 5
				3.29 m			
8	6.61 s	93.1, CH	4, 6, 7, 10	1.06 t (6.5)	15.1, CH_3_	7	7
9	-	152.6, C	-	-	-	-	-
10	-	104.5, C		-	-	-	-
1'	-	121.5, C			-	-	-
2'	6.75 d (1.5)	110.8, CH	1', 3', 4', 6'	-	-	-	-
3'	-	151.2, C		-	-		-
4'	-	136.1, C		-	-	-	-
5'	-	152.2, C		-	-	-	-
6'	6.75 d (1.5)	103.8, CH	1', 2', 4', 5'	-	-	-	-
4-OH	-	-	-	4.95 d (3.5)	-	4	3, 4, 5
5-OOH	-	-	-	4.58 s		-	5, 6
5-OH	12.97 s	-	4, 5, 6, 10	-	-	-	-
3'-OH	9.31 s	-	2, 4	-	-	-	-
4'-OCH_3_	3.70 s	59.9, CH_3_	4'	-	-	-	-
5'-OCH_3_	3.79 s	55.8, CH_3_	5'	-	-	-	-
6-OCH_3_	3.76 s	59.9, CH_3_	6	-	-	-	-
7-OCH_3_	3.88 s	55.9, CH_3_	7	-	-	-	-

**Figure 2:**
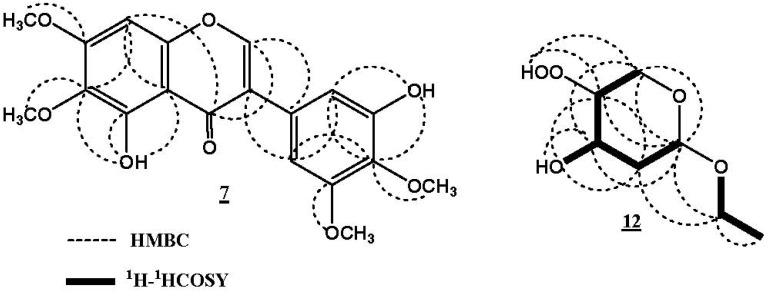
Important ^1^H-^1^H COSY and HMBC correlations of 7 and 12.

The ^13^C-NMR showed nineteen carbon signals, their multiplicities were determined through DEPT, as well as, direct correlations of protons to their respective carbons in the HMQC spectrum. The placement of the methoxy groups at C-4', C-6, C-5', and C-7 was supported by the HMBC spectrum that displayed correlations between 4'-OCH_3_ and C-4'/δ_C_136.1, 6-OCH_3_ and C-6/δ_C_ 132.1, 5'-OCH_3_ and C-5'/δ_C_ 152.2, as well as 7-OCH_3_ and C-7/δ_C_ 154.5 (Figure 2). On the basis of the above evidences, the structure of **7** was elucidated as 3',5-dihydroxy-4',5',6,7-tetramethoxyisoflavone and considered as a new natural product, for which we propose the name irigenin S.

Iriside A (**12**) was isolated as yellow oil. The molecular formula C_7_H_14_O_5_ for **12** was confirmed from the HRFABMS molecular ion peak at *m*/*z* 179.0840 [M+H]^+^, which required one degree of unsaturation. Its IR spectrum displayed an absorption band at 3452 cm^−1^, indicating the presence of a hydroperoxy group [36]. The ^13^C-NMR and HMQC spectra of **12** showed signals for one methyl carbon (δ_C_ 15.1, C-8), three methylenes, two of them which are oxygenated [δ_C_ 63.7 (C-6) and 62.1 (C-7)], and three oxygen-bonded methane carbons at δ_C_ 103.2, 70.9, and 86.9, ascribed to C-2, C-4, and C-5, respectively. The COSY spectrum displayed the presence of two spin systems. The first spin system consists of the triplet methyl signal at δ_H_ 1.06 (*J* = 6.5 Hz, H-8) coupled with the methylene protons at δ_H_ 3.61 and 3.29 indicating the presence of an ethoxy group, which further confirmed by the fragment ion peak at *m*/*z* 150.1503 [M-C_2_H_5_+H]^+^. The second spin system was assigned to a substituted pyran moiety, and confirmed by the fragment ion peak at *m*/*z* 84.0354 [C_5_H_8_O]^+^. This moiety was also validated by the correlations observed in the HMBC spectrum (Figure 2). H-2 showed HMBC correlations with C-4 and C-6 and a four bond coupling to C-5. H-6 correlated with C-2 and C-4 and H-3 with C-5. In the ^1^H-NMR spectrum, the two signals at δ_H_ 4.58 and 4.95 indicated the presence of two hydroxy groups; one for the hydroperoxy group at C-5 and the other for C-4-OH, which were confirmed by the observed COSY and HMBC correlations. The downfield shift of C-5 (δ_C_ 86.9) suggested the presence of a hydroperoxy functional group at C-5 and was confirmed by the fragment ion peaks at *m*/*z* 133.0905 [M-C_2_H_6_O+H]^+^ and 117.0782 [M-C_2_H_6_O_2_+H]^+^ [37]. The connectivity of the ethoxy group at C-2 of the pyran ring was established through the HMBC correlations of H-2 with C-7, H-7 with C-2 and C-3. On the basis of these findings the structure of **12** was unambiguously elucidated as 2-ethoxytetrahydro-5-hydroperoxy-(2*R*,4*R*,5*S*)-2*H*-pyran-4-ol, for which we propose the name iriside A. The relative stereochemistry of **12** was determined by comparing the ^1^H-, ^13^C-NMR chemical shifts, and the coupling constants values with literature [38,39], and the absolute stereochemistry was confirmed using the Mosher procedure [40].

The methanolic extract of *Iris germanica* (MIG) and compounds **5–8**, **10**, **11** were tested for their anti-inflammatory effects using the induced paw edema test. All the tested compounds as well as MIG exhibited potent anti-inflammatory effects; compound **7** showed the highest activity, which was almost similar to that of dexamethasone (Table 2). These results are in accordance with previous studies that attributed the anti-inflammatory activity of flavonoids to the C-2,3 double bond [41] and the presence of a methoxyl group at C-5 and C-7, and the pyran ring [42]. The activity of MIG may be due to the presence of different classes of terpenes and flavonoids. The MIG also showed potent antimicrobial activity against different bacterial and fungal strains (Table 3) at concentrations 250 and 500 µg per disc. It exhibited highest activity at a concentration of 250 µg per disc against *S. aureus*, *S. marcescens*, *E. coli*, *C. albicans*, and *A. flavus*. This observed anti-microbial effects of the extract might be due to the presence of phenolic constituents in the plant.

**Table 2:** Effect of injection of dexamethasone, MIG, and compounds **5–8**, **10**, and **11** on formalin-induced rat paw edema.

Groups n = 6	Dose mg/kg	Paw Edema Thickness (mm)			
		1 h	2 h	4 h	24 h
Inflamed control		3.10 ± 0.18	3.36 ± 0.02	3.50 ± 0.13	3.40 ± 0.11
Inflamed + dexamethasone	10	1.95 ± 0.09 *	1.83 ± 0.11 *	1.79 ± 0.06 *	1.70 ± 0.08 *
Inflamed + MIG	50	2.80 ± 0.25 *	2.65 ± 0.03 *	2.40 ± 0.11 *	2.20 ± 0.09 *
	100	2.60 ± 0.32 *	2.30 ± 0.18 *	2.15 ± 0.14 *	2.08 ± 0.07 *
Inflamed + **5**	10	2.40 ± 0.11 *	2.20 ± 0.09 *	1.98 ± 0.08 *	1.90 ± 0.08 *
Inflamed + **6**	10	2.10 ± 0.13 *	2.01 ± 0.18 *	1.95 ± 0.14 *	1.86 ± 0.07 *
Inflamed + **7**	10	1.98 ± 0.12 *	1.85 ± 0.14 *	1.78 ± 0.06 *	1.72 ± 0.04 *
Inflamed + **8**	10	2.04 ± 0.09 *	2.01 ± 0.11 *	1.90 ± 0.04 *	1.78 ± 0.07 *
Inflamed + **10**	10	2.50 ± 0.12 *	2.30 ± 0.11 *	2.10 ± 0.09 *	2.01 ± 0.09 *
Inflamed + **11**	10	2.06 ± 0.09 *	2.01 ± 0.18 *	1.93 ± 0.13 *	1.86 ± 0.07 *

* Significant different from inflamed control group at *p* < 0.01.

**Table 3:** Antimicrobial activity of MIG.

		Inhibition zone in mm						
Extract		**Bacterial Strains**					**Fungal Strain**	
	Conc. µg/disc	*B. cereus* (AUMC No. B-52)	*S. aureus* (AUMC No. B-54)	*E. coli* (AUMC No. B-53)	*P. aeruginosa* (AUMC No. B-73)	*S. marcescens* (AUMC No. B-55)	A. *flavus* (AUMC No. 1276)	*C. albicans* (AUMC No. 226)
MIG	250	21	18	12	12	24	19	12
	500	39	32	22	19	36	37	22
Chloroamphenicol ^a^	250	55	43	27	20	43		-
Clotriamazole ^b^	250	-	-	-	-	-	43	25

AUMC: Assiut University Mycology Center. ^a^ Positive control for antibacterial activity; ^b^ Positive control for antifungal activity.

## 3. Experimental

### 3.1. General Procedures

Optical rotation was measured on a Perkin-Elmer Model 341 LC polarimeter. Melting points were determined using an Electrothermal 9100 Digital Melting Point apparatus (Electrothermal Engineering Ltd., Essex, England). HRESI and FABMS were recorded on a LTQ Orbitrap and an API 2000 (ThermoFinnigan, Bremen, Germany) mass spectrometers, respectively. Low resolution mass spectra were determined using a Finnigan MAT TSQ-7000 mass spectrometer. UV spectra were recorded in absolute MeOH on a Shimadzu 1601 UV/VIS spectrophotometer. The IR spectra were measured on a Shimadzu Infrared-400 spectrophotometer (Kyoto, Japan). 1D and 2D NMR spectra (chemical shifts in ppm, coupling constants in Hz) were recorded on Bruker BioSpin GmbH 500 MHz Ultrashield spectrometer using standard Bruker software and DMSO-*d*_6_, CDCl_3_, and C_5_D_5_N as solvents, with TMS as the internal reference. Solvents were distilled prior spectroscopic measurements. Column chromatographic separations were performed on silica gel 60 (0.04-0.063 mm), RP-18 (0.04–0.063 mm, Merck), and Sephadex LH-20 (0.25–0.1 mm, Merck). TLC was performed on precoated TLC plates with silica gel 60 F_254_ (layer thickness 0.2 mm, Merck). The solvent systems used for TLC analyses were CHCl_3_-MeOH (97:3, solvent system I), CHCl_3_-MeOH (90:10, solvent system II), and CHCl_3_-MeOH (85:15, solvent system III). The compounds were detected by UV absorption at λ_max_ 255 and 366 nm followed by spraying with anisaldehyde/H_2_SO_4_ reagent and heating at 110 °C for 1–2 min. 

### 3.2. Plant Material

The rhizomes of *Iris germanica* L. were collected in April 2007 from plants growing at the botanical garden of the Faculty of Agriculture, Assiut University. The plant material was kindly identified by A. Fayed, Professor of Plant Taxonomy, Faculty of Science, Assiut University. A voucher specimen was deposited in herbarium of the Department of Pharmacognosy, Faculty of Pharmacy, Al-Azhar University, Assiut under the registration number IG-1-2007.

### 3.3. Extraction and Isolation

The air-dried powdered rhizomes of *Iris germanica* L. (320 g) were extracted with 70% methanol (6 × 2 L) at room temperature to afford a dark brown residue (18.1 g). The total methanolic extract was subjected to normal phase vacuum liquid chromatography (VLC) using *n*-hexane, CHCl_3_, EtOAc, and MeOH to afford four fractions; *n*-hexane IG-1 (3.5 g), CHCl_3_ IG-2 (4.0 g), EtOAc IG-3 (3.2 g), and MeOH IG-4 (5.3 g) fractions. Fraction IG-2 (4.0 g) was subjected to VLC by gradient elution using *n*-hexane-EtOAc as solvents, and four subfractions were obtained; IG-2-A (0.85 g), IG-2-B (0.90 g), IG-2-C (0.65 g), and IG-2-D (0.75 g). Subfraction IG-2-A (0.85 g) was chromatographed over silica gel column (140 g × 50 × 3 cm) using a *n*-hexane-EtOAc gradient to afford compounds **1** (40 mg), **2** (15 mg), and **3** (10 mg). Subfraction IG-2-B (0.90 g) was subjected to silica gel column chromatography (150 g × 50 × 3 cm) using CHCl_3_-MeOH (95:5) as an eluent to give compounds **4** (20 mg) and **5** (35 mg). A Sephadex LH-20 column chromatorgraphy (150 g × 100 × 5 cm) of subfraction IG-2-C (0.65 g) using MeOH:CHCl_3_ (9:1) give impure compounds **6**, **7**, and **8** which were further purified by repeated chromatography on a silica gel column using a CHCl_3_-MeOH gradient to afford pure compounds **6** (25 mg), **7** (30 mg), and **8** (55 mg). Compound **9** (100 mg) was obtained as a white amorphous powder upon crystallization of fraction IG-2-D using MeOH. Fraction IG-3 (3.2 g) was subjected to Sephadex LH-20 chromatography using MeOH as eluent to afford four subfractions IG-3-A (0.45 g), IG-3-B (0.51 g), IG-3-C (0.75 g), and IG-3-D (0.68 g). Subfraction IG-3-A was chromatographed over a silica gel column (100 g × 50 × 3 cm) using a CHCl_3_-MeOH gradient to give compound **10** (35 mg). RP-18 column chromatography (100 g × 50 × 3 cm) of subfraction IG-3-B using a MeOH-H_2_O gradient give compounds **11** (25 mg) and **12** (40 mg).

### 3.4. Spectral Data

*Stigmasterol* (**1**). White needles, m.p. 169–170 °C, EIMS: *m*/*z* 412 [M]^+^. ^1^H-NMR (CDCl_3_, 500 MHz): δ_H_ 3.55 (m, H-3), 5.38 (brs, H-6), 0.69 (s, H-18),1.02 (s, H-19), 0.93 (d, *J* = 6.1 Hz, H-21), 5.18 (dd, *J* = 15.4, 8.0 Hz, H-22), 5.11 (dd, *J* = 15.4, 8.0 Hz, H-23), 0.84 (d, *J* = 7.3 Hz, H-26), 0.84 (d, *J* = 7.3 Hz, H-27), 0.86 (t, *J* = 7.1 Hz, H-29). ^13^C-NMR (CDCl_3_, 125 MHz): δ_C_ 37.2 (C-1), 31.8 (C-2), 71.9 (C-3), 39.4 (C-4), 140.8 (C-5), 121.8 (C-6), 29.1 (C-7), 31.9 (C-8), 50.1 (C-9), 35.9 (C-10), 20.6 (C-11), 41.0 (C-12), 42.2 (C-13), 56.1 (C-14), 23.3 (C-15), 24.6 (C-16), 56.8 (C-17), 11.9 (C-18), 18.9 (C-19), 39.8 (C-20), 20.9 (C-21), 138.4 (C-22), 129.3 (C-23), 51.2 (C-24), 31.9 (C-25), 19.4 (C-26), 19.8 (C-27), 26.7 (C-28), 12.0 (C-29).

*α-Irone* (**2**). Colorless oil. EIMS: *m/z* 206 [M]^+^. ^1^H-NMR (CDCl_3_, 500 MHz): δ_H_ 2.33 (s, H-1), 6.10 (d, *J* = 15.8 Hz, H-3), 6.66 (dd, *J* = 15.8, 11.0 Hz, H-4), 2.59 (m, H-5), 5.54 (brs, H-7), 1.98, 1.78 (2H each m, H-8), 1.52 (m, H-9), 0.88 (s, H-11), 1.63 (s, H-12), 0.73 (s, H-13), 0.88 (d, *J* = 6.5 Hz, H-14). ^13^C-NMR (CDCl_3_, 125 MHz): δ_C_ 27.0 (C-1), 198.3 (C-2), 133.6 (C-3), 149.6 (C-4), 56.0 (C-5), 132.0 (C-6), 123.1 (C-7), 31.8 (C-8), 37.9 (C-9), 35.8 (C-10), 15.4 (C-11), 23.0 (C-12), 15.0 (C-13), 26.6 (C-14).

γ-*Irone* (**3**). Colorless oil. EIMS: *m/z* 206 [M]^+^. ^1^H-NMR (CDCl_3_, 500 MHz): δ_H_ 2.37 (s, H-1), 6.15 (d, *J* = 16.0 Hz, H-3), 6.95 (dd, *J* = 16.0, 10.5 Hz, H-4), 2.85 (m, H-5), 2.36, 2.15 (2H each m, H-7), 1.56, 1.25 (2H each m, H-8), 1.45 (m, H-9), 0.88 (s, H-11), 4.82, 4.45 (2 H each brs, H-12), 0.74 (s, H-13), 0.88 (d, *J* = 6.5 Hz, H-14). ^13^C-NMR (CDCl_3_, 125 MHz): δ_C_ 27.3 (C-1), 198.3 (C-2), 134.3 (C-3), 147.3 (C-4), 57.8 (C-5), 148.8 (C-6), 36.3 (C-7), 31.9 (C-8), 41.9 (C-9), 38.8 (C-10), 15.9 (C-11), 108.7 (C-12), 14.1 (C-13), 27.7 (C-14).

*3-Hydroxy-5-methoxyacetophenone* (**4**). Colorless crystals, (MeOH), R*_f_* = 0.78 (solvent system I), m.p. 188–189 °C. EIMS: *m*/*z* 166 [M]^+^. ^1^H-NMR (CDCl_3_, 500 MHz): δ_H_ 7.55 (d, *J* = 1.5 Hz, H-2), 6.97 (d, *J* = 1.5 Hz, H-4), 7.55 (d, *J* = 1.5 Hz, H-6), 2.58 (s, H-8), 6.22 (s, 3-OH), 3.97 (s, 5-OCH_3_). ^13^C-NMR (CDCl_3_, 125 MHz): δ_C_ 130.2 (C-1), 124.1 (C-2), 150.4 (C-3), 113.8 (C-4), 146.6 (C-5), 109.7 (C-6), 196.9 (C-7), 26.2 (C-8), 56.1 (5-OCH_3_).

*Irilone* (**5**). Yellow needles (MeOH), m.p. 233–234 °C. EIMS: 298 [M]^+^. λ_max_ 205, 261, and 324 nm. ^1^H-NMR (DMSO-*d*_6_, 500 MHz): δ_H_ 8.44 (s, H-2), 6.90 (s, H-8), 7.40 (2 H, d, *J* = 8.0 Hz, H-2', 6'), 6.83 (2 H, d, *J* = 8.0 Hz, H-3', 5'), 6.19 (s, -O-CH_2_-O-), 9.65 (s, 4'-OH), 12.94 (s, 5-OH). ^13^C-NMR (DMSO-*d*_6_, 125 MHz): δ_C_ 154.7 (C-2), 122.1 (C-3), 180.9 (C-4), 141.3 (C-5), 129.5 (C-6), 153.9 (C-7), 89.5 (C-8), 152.9 (C-9), 107.4 (C-10), 120.9 (C-1'), 130.2 (C-2', 6'), 115.1 (C-3', 5'), 157.5 (C-4'), 102.8 (-O-CH_2_-O-).

*Irisolidone* (**6**). Yellow amorphous powder. EIMS: *m/z* 314 [M]^+^. UV (MeOH) λ*_max_* nm: 207, 264, and 328. ^1^H-NMR (DMSO-*d*_6_, 500 MHz): δ_H_ 7.88 (s, H-2), 6.59 (s, H-8), 7.47 (d, *J* = 8.5 Hz, H-2', 6'), 6.99 (d, *J* = 8.5 Hz, H-3', 5'), 3.86 (s, 4'-OCH_3_), 4.05 (s, 6-OCH_3_), 13.15 (s, 5-OH). ^13^C-NMR (DMSO-*d*_6_, 125 MHz): δ_C_ 153.5 (C-2), 123.1 (C-3), 181.4 (C-4), 152.8 (C-5), 130.4 (C-6), 152.7 (C-7), 93.2 (C-8), 155.3 (C-9), 106.4 (C-10), 122.9 (C-1'), 130.2 (C-2', 6'), 159.7 (C-4'), 114.1 (C-3', 5'), 55.4 (4'-OCH_3_), 60.9 (6-OCH_3_).

*Irigenin S* (**7**). Yellow needles (MeOH), R*_f_* = 0.47 (solvent system II), m.p. 192–193 °C. UV (MeOH) λ*_max_* nm: 201, 265, and 323. IR ν*_max_* (cm^−1^): 3437, 2956, 1651, and 1065. HRESIMS: *m/z* 375.1002 (calcd for C_19_H_19_O_8_ 375.1001). NMR spectral data, see Table 1.

*Irigenin* (**8**). Yellow needles (MeOH), m.p. 185–186 °C. UV (MeOH) λ*_max_* nm: 203, 263, and 323 nm. EIMS: *m*/*z* 360 [M]^+^. ^1^H-NMR (DMSO-*d*_6_, 500 MHz): δ_H_ 8.40 (s, H-2), 6.52 (s, H-8), 6.72 (d, *J* = 1.5 Hz, H-2'), 6.71 (d, *J* = 1.5 Hz, H-6'), 3.70 (s, 4'-OCH_3_), 3.79 (s, 5'-OCH_3_), 3.76 (s, 6-OCH_3_), 9.31 (s, 3'-OH), 13.01 (s, 5-OH), 10.90 (s, 7-OH). ^13^C-NMR (DMSO-*d*_6_, 125 MHz): δ_C_ 154.8 (C-2), 126.0 (C-3), 180.3 (C-4), 153.3 (C-5), 131.4 (C-6), 157.5 (C-7), 93.9 (C-8), 152.8 (C-9), 104.8 (C-10), 121.7 (C-1'), 110.3 (C-2'), 150.2 (C-3'), 136.3 (C-4'), 152.6 (C-5'), 104.4 (C-6'), 59.9 (4'-OCH_3_), 55.7 (5'-OCH_3_), 59.9 (6-OCH_3_).

*Stigmasterol-3-O-β-glucoside* (**9**). White amorphous powder. FABMS: *m*/*z* 575 [M+H]^+^. ^1^H-NMR (C_5_D_5_N, 500 MHz): δ_H_ 3.97 (m, H-3), 0.62 (s, H-18), 1.21 (s, H-19), 0.96–0.81 (methyl groups), 5.06 (d, *J* = 7.6 Hz, H-1'), 4.58–3.86 (m, sugar protons). ^13^C-NMR (C_5_D_5_N, 125 MHz): δ_C_ 38.0 (C-1), 31.1 (C-2), 78.9 (C-3), 40.4 (C-4), 141.4 (C-5), 122.4 (C-6), 32.7 (C-7), 32.5 (C-8), 50.8 (C-9), 37.4 (C-10), 21.8 (C-11), 39.5 (C-12), 43.0 (C-13), 57.3 (C-14), 25.0 (C-15), 23.7 (C-16), 56.7 (C-17), 11.6 (C-18), 19.9 (C-19), 39.8 (C-20), 19.5 (C-21), 133.6 (C-22), 129.8 (C-23), 46.5 (C-24), 29.9 (C-25), 19.7 (C-26), 20.5 (C-27), 24.5 (C-28), 12.5 (C-29), 103.0 (C-1'), 72.2 (C-2'), 75.8 (C-3'), 68.7 (C-4'), 78.7 (C-5'), 63.3 (C-6').

*Irilone 4'-O-β-D-glucopyranoside* (**10**). Yellow amorphous powder. UV (MeOH) λ*_max_* nm: 203, 271, and 337 nm. FABMS: *m/z* 461 [M+H]^+^, 299 [M-162 (hexose unit)+H]^+^. ^1^H-NMR (DMSO-*d*_6_, 500 MHz): δ_H_ 8.51 (s, H-2), 7.01 (s, H-8), 7.34 (2 H, d, *J* = 8.5 Hz, H-2', 6'), 6.81 (2 H, d, *J* = 8.5 Hz, H-3', 5'), 6.19 (s, -O-CH_2_-O-), 13.42 (s, 5-OH), 4.98 (d, *J* = 6.7 Hz, H-1”). ^13^C-NMR (DMSO-*d*_6_, 125 MHz): δ_C_ 155.4 (C-2), 123.0 (C-3), 181.0 (C-4), 139.8 (C-5), 129.4 (C-6), 153.5 (C-7), 93.6 (C-8), 151.7 (C-9), 106.9 (C-10), 120.9 (C-1'), 130.2 (C-2', 6'), 114.8 (C-3', 5'), 157.9 (C-4'), 102.6 (-O-CH_2_-O-), 100.9 (C-1”), 73.6 (C-2”), 77.4 (C-3”), 69.8 (C-4”), 76.7 (C-5”), 60.6 (C-6”).

*Iridin* (**11**). Yellow needles, m.p. 210–211 °C. UV (MeOH) λ*_max_* nm: 203, 263, and 335 nm. FABMS: *m/z* 523 [M+H]^+^, 361 [M-162 (hexose unit)+H]^+^. ^1^H-NMR (DMSO-*d*_6_, 500 MHz): δ_H_ 8.51 (s, H-2), 6.70 (s, H-8), 6.91 (brs, H-2'), 6.74 (brs, H-6'), 3.71 s (s, 4'-OCH_3_), 3.80 (s, 5'-OCH_3_), 3.78 (s, 6-OCH_3_), 9.30 (s, 3'-OH), 12.95 (s, 5-OH), 5.11 (d, *J* = 6.7 Hz, H-1”). ^13^C-NMR (DMSO-*d*_6_, 125 MHz): δ_C_ 155.4 (C-2), 125.9 (C-3), 180.5 (C-4), 152.9 (C-5), 132.5 (C-6), 156.6 (C-7), 94.0 (C-8), 152.4 (C-9), 104.5 (C-10), 122.0 (C-1'), 110.3 (C-2'), 150.3 (C-3'), 136.4 (C-4'), 152.9 (C-5'), 104.5 (C-6'), 100.1 (C-1'), 73.1 (C-2'), 77.3 (C-3”), 69.6 (C-4”), 76.7 (C-5”), 60.3 (C-6”).

*Iriside A* (**12**). Yellow oil, R*_f_* = 0.42 (solvent system III), [α]_D_ +72.6° (*c* 0.3, MeOH). IR ν*_max_* (cm^−1^): 3452, 2945, 1455, 1103, 983. HRFABMS: *m*/*z* 179.0840 (*calc.* for C_7_H_15_O_5_, 179.0841). NMR spectral data, see Table 1.

### 3.5. Mosher Procedure *[40]*

Four mg of compound **12** was dissolved in pyridine-*d*_5_ (0.7 mL) and transferred to NMR tubes. The ^1^H-NMR spectrum of **12** was measured prior to adding 5 μL of (*R*)-MTPA-Cl and (*S*)-MTPA-Cl reagent (Fluka, Germany), respectively. The tubes were shaken thoroughly and were allowed to stand at room temperature for 72 h. The reaction was monitored by ^1^H-NMR spectroscopy after every 24 h. 

### 3.6. Biological Studies

#### 3.6.1. Anti-Inflammatory Activity

Hind paw edema (skin edema) was induced by 4% formalin solution injected into the subplantar region of the left hind paw [42]. Adult male albino rats 100–120 g (purchased from the Animal House, Pharmacology Department, Faculty of Medicine, Assiut University). The inflamed animals were divided randomly into ten groups (six in each group), inflamed control group, inflamed treated with dexamethasone (at a dose of 10 mg/kg subcutaneously), six groups of inflamed animals were treated with the tested compounds individually (at a dose of 10 mg/kg subcutaneously), and two groups were treated with MIG at doses of 50 and 100 mg/kg subcutaneously (the plant extract was dissolved in sterile distilled water). The change in paw thickness in all tested animals was measured at 1, 2, 4, and 24 h after formalin solution injection. The data were expressed as mean ± S.D. using the Student *t* test (Table 2).

#### 3.6.2. Antimicrobial Assay

The procedure was carried out as previously described [43]. The antibacterial and antifungal activities were evaluated using the agar plate diffusion assay. Susceptibility discs (5.5 mm) were impregnated with solution of each of extract at concentrations 250 and 500 µg per disc. The discs were dried and placed on agar plates inoculated with the test bacterial strains: *Bacillus cereus* (AUMC No. B-52), *Staphylococcus aureus* (AUMC No. B-54), *Escherichia coli* (AUMC No. B-53), *Pseudomonas aeruginosa* (AUMC No. B-73), and *Serratia marcescens* (AUMC No. B-55), and the fungal strains: *Candida albicans* (AUMC No. 418), *Geotrichum candidum* (AUMC No. 226), *Trichophyton rubrum* (AUMC No. 1804), *Fusarium oxysporum* (AUMC No. 209), *Scopulariopsis brevicaulis* (AUMC No. 729), and *Aspergillus flavus* (AUMC No. 1276). Each plate was inoculated with a single organism and the test was run in duplicates. The plates were incubated at 37 °C and checked for inhibition zones after 24 h for bacteria and after 48 h for fungi. Chloroamphenicol and clotrimazole at concentration 250 µg per disc were used as positive reference standards for antibacterial and antifungal activities, respectively.

## 4. Conclusions

Two new compounds; irigenin S (**7**) and iriside A (**12**), were isolated from the rhizomes of *I. germanica* L. growing in Egypt along with ten known compounds. The methanolic extract (MIG) and the isolated flavonoids exhibited potent anti-inflammatory effects. Compound **7** showed activity similar to that of dexamethasone. The MIG showed highest anti-microbial effect against *S. aureus*, *S. marcescens*, *E. coli*, *C. albicans*, and *A. flavus*.
